# Sex Disparities in Outcomes After Minimally Invasive Direct CABG for Single-Vessel Disease: A Propensity Score-Matched Analysis

**DOI:** 10.3390/jcm15176821

**Published:** 2026-09-03

**Authors:** Lukman Amanov, Arian Arjomandi Rad, Sadeq Ali-Hasan-Al-Saegh, Jawad Salman, Fabio Ius, Abdullah Tahir, Thanos Athanasiou, Saeed Torabi, Stefan Rümke, Bastian Schmack, Arjang Ruhparwar, Alina Zubarevich, Alexander Weymann

**Affiliations:** 1 Department of Cardiothoracic, Transplantation and Vascular Surgery, Hannover Medical School, Carl-Neuberg-Straße 1, 30625 Hannover, Germany; lukman.tm@gmail.com (L.A.); al-saegh.sadeq@mh-hannover.de (S.A.-H.-A.-S.); salman.jawad@mh-hannover.de (J.S.); ius.fabio@mh-hannover.de (F.I.); schmack.bastian@mh-hannover.de (B.S.); ruhparwar.arjang@mh-hannover.de (A.R.); weymann.alexander@gmail.com (A.W.); 2 Department of Cardiothoracic Surgery, Oxford Heart Centre, John Radcliffe Hospital, Oxford University Hospitals NHS Foundation Trust, Oxford OX3 9DU, UK; 3 Department of Trauma and Orthopedics, University Hospitals of North Midlands NHS Trust, Stoke-on-Trent ST4 6QG, UK; abdullah.tahir3@nhs.net; 4 Department of Surgery and Cancer, Imperial College London, London SW7 2AZ, UK; t.athanasiou@imperial.ac.uk; 5 Department of Anesthesiology and Intensive Care Medicine, Medical Faculty of Cologne University, University Hospital of Cologne, 50937 Cologne, Germany; saeed.torabi@uk-koeln.de

**Keywords:** minimally invasive direct coronary artery bypass, MIDCAB, sex, gender, propensity score matching, left anterior descending artery, long-term survival, coronary revascularization

## Abstract

**Background:** Female sex is widely regarded as an independent risk factor for adverse outcomes after conventional coronary artery bypass grafting (CABG) and is incorporated as a risk variable in EuroSCORE II. Whether this disadvantage persists in the setting of minimally invasive direct coronary artery bypass (MIDCAB), in which sternotomy and cardiopulmonary bypass are avoided, remains insufficiently characterised. We assessed sex-specific short- and long-term outcomes after MIDCAB in a single-centre cohort with extended follow-up. **Methods:** We retrospectively analysed 350 consecutive patients who underwent MIDCAB at Hannover Medical School between July 1999 and April 2025 (follow-up to April 2025). Eligibility criteria and heart team-applied exclusion criteria (prior left thoracotomy, unfavourable LAD anatomy, prohibitive respiratory reserve, hostile chest wall, active endocarditis, or haemodynamic instability requiring on-pump revascularization) are detailed in the Methods. Females (*n* = 102) and males (*n* = 248) were compared before and after 1:1 propensity score matching using greedy nearest-neighbour matching with a caliper of 0.2 × SD of the logit propensity score. The primary endpoint was all-cause long-term mortality; secondary endpoints included perioperative complications and in-hospital outcomes. Long-term survival was assessed by Kaplan–Meier analysis and multivariable Cox proportional hazards regression performed in the full unmatched cohort. A pre-specified subgroup analysis of long-term survival by coronary disease pattern (single-vessel vs. multivessel disease) was also performed. **Results:** Matching produced 100 female–male pairs with excellent covariate balance (all standardized mean differences < 0.20). MIDCAB was completed without intraoperative conversion in all patients. Thirty-day mortality was 0% in both sexes; no postoperative stroke or new requirement for dialysis occurred. New-onset atrial fibrillation (3.0% vs. 1.0%, *p* = 0.621), length of intensive care unit stay (median 1 day in both groups), and hospital length of stay (median 8 days in both groups) were comparable between females and males. Re-exploration for bleeding was numerically more frequent in women (5.0% vs. 0.0%; Newcombe 95% CI for the risk difference +0.3 to +11.2%; Fisher’s exact *p* = 0.059). At a median follow-up of 19.0 years (IQR 11.8–23.9), all-cause mortality was identical (12.0% vs. 12.0%, *p* = 1.000; log-rank *p* = 0.703). In multivariable Cox regression in the full unmatched cohort, female sex was not associated with long-term mortality (adjusted HR 0.80, 95% CI 0.38–1.70, *p* = 0.560); only advancing age emerged as a strong independent predictor (HR 1.10 per year, 95% CI 1.05–1.15, *p* < 0.001), with EuroSCORE II approaching significance (HR 1.67 per unit, 95% CI 1.00–2.82, *p* = 0.052). Long-term survival in patients with multivessel disease (20-year Kaplan–Meier 90.9%) was equivalent to that in single-vessel disease (92.3%; log-rank *p* = 0.94). **Conclusions:** In this propensity-matched analysis with two decades of follow-up, MIDCAB conferred equivalent perioperative safety and long-term survival in women and men. Female sex was not an independent predictor of adverse outcome. These findings support MIDCAB as a sex-neutral revascularization strategy for single-vessel and LAD-predominant coronary artery disease in the very low-risk, appropriately selected population studied, and are consistent with the most recent published MIDCAB literature.

## 1. Introduction

Coronary artery disease remains the leading cause of death worldwide and a major contributor to morbidity in both women and men. Coronary artery bypass grafting (CABG) has been the cornerstone of surgical revascularization for more than five decades, and its survival benefit—particularly when the left internal mammary artery (LIMA) is anastomosed to the left anterior descending (LAD) coronary artery—remains unmatched by any alternative therapy for selected anatomical subsets [1,2]. However, conventional CABG via median sternotomy with cardiopulmonary bypass is associated with substantial perioperative trauma, including risks of sternal wound complications, neurocognitive injury, transfusion requirements and prolonged recovery, particularly in higher-risk subgroups.

Sex-based differences in outcome after conventional CABG have been the subject of intense debate for more than three decades. Multiple large registries and meta-analyses have demonstrated that women undergoing CABG present at an older age with a greater burden of comorbidities—including diabetes mellitus, arterial hypertension and renal dysfunction—and more frequent urgent or emergent indications than men [3,4]. Consequently, women have repeatedly been shown to suffer higher rates of early mortality and perioperative complications, an observation reflected in widely used risk-prediction tools: female sex is incorporated as an independent risk variable in EuroSCORE II [5]. Whether female sex truly represents an independent biological risk factor, or whether it is largely a marker of an unfavourable baseline risk profile, remains controversial. After adjustment for baseline comorbidities and body size, several propensity-matched analyses have not demonstrated an independent effect of sex on early or late mortality, suggesting that confounding rather than biology may explain much of the apparent disparity [6].

Minimally invasive direct coronary artery bypass (MIDCAB), introduced by Calafiore and colleagues in 1996 [7], addresses isolated LAD disease or the LAD component of multivessel disease in the context of hybrid revascularization through a small left anterolateral thoracotomy and off-pump grafting of the LIMA to the LAD. By avoiding both sternotomy and cardiopulmonary bypass, MIDCAB substantially reduces perioperative morbidity, and the LIMA-LAD graft demonstrates durable long-term patency exceeding 90% at 10–15 years [1,2,8]. These attributes might be expected to disproportionately benefit subgroups in whom conventional CABG carries the highest relative risk: older patients, frail patients, and possibly women.

Data on sex-specific outcomes after MIDCAB remain limited. Existing series vary in size, follow-up duration, and reported findings: Gofus and colleagues found longer operative times, more transfusions and more wound complications in women but no excess mortality [9]; Friedrich and colleagues reported equivalent in-hospital outcomes and, after propensity matching, even improved long-term survival in women [10]; Zhao and colleagues recently confirmed comparable mortality and major adverse cardiovascular events between sexes in a propensity-matched analysis of single-vessel disease [11]; and the most recent 20-year series by Comanici and colleagues again demonstrated equivalent long-term survival between the sexes [12]. Given the increasing adoption of minimally invasive coronary revascularization and the importance of sex-informed clinical decision-making, robust analyses with long follow-up are needed.

The present study was designed to evaluate sex-specific outcomes after MIDCAB performed for single-vessel or LAD-predominant coronary artery disease in a single-centre cohort with a median follow-up approaching two decades. To minimise confounding by baseline differences, we applied 1:1 propensity score matching and assessed perioperative complications, in-hospital outcomes and long-term all-cause mortality. We further used multivariable Cox regression to determine whether female sex is an independent predictor of long-term mortality after MIDCAB.

## 2. Materials and Methods

### 2.1. Study Design and Patient Population

This was a retrospective, single-centre, observational cohort study of consecutive patients who underwent MIDCAB at Hannover Medical School between July 1999 and April 2025, with follow-up extending to April 2025. Eligible patients were adults (≥18 years) in whom MIDCAB was performed for single-vessel coronary artery disease (stenosis of the LAD) or as the surgical component of a hybrid revascularization strategy for multivessel disease with an LAD lesion. The decision to perform MIDCAB was individualised on the basis of coronary anatomy, comorbidity profile, multidisciplinary heart team consensus and patient preference, in accordance with the 2018 ESC/EACTS guidelines on myocardial revascularization [13].

Exclusion criteria applied by the multidisciplinary heart team in selecting patients for MIDCAB were: (i) prior left thoracotomy or significant left pleural adhesions precluding a repeat left-sided approach; (ii) anatomically unfavourable LAD (very small calibre, extensive calcification, entirely intramyocardial course, or exclusively distal disease); (iii) inability to tolerate single-lung ventilation because of severe respiratory reserve limitation; (iv) severe pectus deformity or extensive post-radiotherapy fibrosis producing an anatomically hostile left hemithorax; (v) active endocarditis; and (vi) haemodynamic instability requiring immediate on-pump revascularization. No patient was excluded from the final analysis on the basis of missing data, adverse outcome, or protocol violation.

The study was conducted in accordance with the Declaration of Helsinki. Ethical review and approval were waived for this retrospective, non-interventional study because it used fully anonymized patient data. In accordance with German regulations, including §15 of the professional code of conduct of the German Medical Association and the corresponding regulations of the State Medical Chambers, retrospective studies based exclusively on anonymized data do not require prior ethics committee approval.

### 2.2. Surgical Technique

All MIDCAB procedures were performed under general anaesthesia with single-lumen endotracheal intubation, in the technique originally described by Calafiore and colleagues [7]. Patients were positioned supine with slight elevation of the left hemithorax. A small (approximately 5–8 cm) left anterolateral thoracotomy was performed in the fourth or fifth intercostal space, tailored to coronary anatomy and operator preference. The LIMA was harvested under direct vision using a dedicated soft-tissue retractor, without rib resection. After systemic heparinisation, the LAD was exposed and stabilised with an epicardial stabiliser. The LIMA-to-LAD anastomosis was constructed off-pump on the beating heart using running monofilament suture. Intraoperative graft patency was verified by direct inspection and, where indicated, by transit-time flow measurement. Intraoperative conversion to conventional sternotomy or cardiopulmonary bypass was pre-specified as a distinct outcome. No intraoperative conversion occurred in the 350 consecutive patients in the present cohort (0/350; exact 95% CI 0.00–1.05%).

### 2.3. Data Collection and Definitions

Demographic, clinical, operative and outcome data were extracted from the institutional electronic medical record and prospectively maintained surgical database. Preoperative variables included age, sex, EuroSCORE II [5], left ventricular ejection fraction (LVEF), urgency of operation (elective, urgent or emergency), coronary artery disease pattern (single-, two-, or three-vessel involvement), recent myocardial infarction (<90 days), ST-elevation and non-ST-elevation myocardial infarction, prior percutaneous coronary intervention (PCI), smoking status, diabetes mellitus (non-insulin- and insulin-dependent), arterial hypertension, hyperlipidemia, renal impairment and preoperative dialysis.

Renal impairment was defined as an estimated glomerular filtration rate < 60 mL/min/1.73 m^2^ by the CKD-EPI equation (KDIGO CKD stage ≥ G3a) or dependence on chronic dialysis at the time of surgery. LVEF categories followed the EuroSCORE II definitions (>50% good, 31–50% moderate, 21–30% poor, ≤20% very poor). Preoperative valvular function was assessed by transthoracic echocardiography; moderate or greater mitral (MVI > II) and tricuspid regurgitation (TVI > II) were recorded as discrete variables. NYHA functional class, body mass index, and alcohol-use history were not systematically recorded as discrete fields in the institutional database across the study period and are therefore not reported; the absence of these variables is acknowledged as a limitation (Section 4.6).

EuroSCORE II was published by Nashef and colleagues in 2012 [5]. For patients operated before this publication (i.e., between July 1999 and 2011, representing approximately the first two-thirds of the study period), EuroSCORE II was calculated retrospectively from the prospectively recorded preoperative variables at the time of manuscript preparation using the published EuroSCORE II algorithm. All variables required by the score were prospectively recorded in the surgical database and are therefore amenable to retrospective algorithmic scoring. This is standard practice in retrospective cohort studies with extended follow-up and permits a single uniform risk metric to be applied across the entire cohort.

Intraoperative variables included operative time, intraoperative red blood cell (RBC) and platelet transfusion, and conversion to conventional CABG. Postoperative in-hospital endpoints included the length of intensive care unit (ICU) stay, duration of mechanical ventilation, hospital length of stay, new-onset atrial fibrillation (NOAF), re-exploration for bleeding, new postoperative dialysis, stroke, postoperative myocardial infarction, peak creatine kinase (CK), CK-MB and troponin, postoperative RBC transfusion in the ICU, and the need for postoperative coronary angiography. The primary endpoint was all-cause long-term mortality. Secondary long-term endpoints were 30-day mortality (death within 30 days of surgery), long-term myocardial infarction and the patency of the LIMA-LAD anastomosis among patients who underwent follow-up coronary angiography.

### 2.4. Follow-Up

Follow-up was obtained from outpatient clinic visits, written correspondence with referring cardiologists and general practitioners, and direct telephone contact with patients or relatives where necessary. Mortality status was cross-checked against national registry data. Follow-up duration was calculated from the date of surgery to the date of death or last contact. Follow-up was 100% complete for the primary endpoint of all-cause mortality.

### 2.5. Statistical Analysis

Continuous variables were assessed for normality by visual inspection and Shapiro–Wilk testing and are reported as mean ± standard deviation (SD) or median with interquartile range (IQR), as appropriate. Categorical variables are reported as counts and percentages. Between-sex comparisons used Welch’s *t*-test or the Mann–Whitney U test for continuous variables, and the chi-square (χ^2^) or Fisher’s exact test for categorical variables, with the latter applied when expected cell counts were below five.

To minimise confounding by baseline differences between sexes, a propensity score for female sex was estimated using multivariable logistic regression. Variables entered into the propensity model were selected a priori as clinically relevant determinants of perioperative risk and long-term outcome, and included age, EuroSCORE II, LVEF, single-, two- and three-vessel coronary artery disease, recent myocardial infarction, prior PCI, smoking, diabetes mellitus, arterial hypertension, hyperlipidemia, renal impairment and non-elective status. Predictors were standardised prior to model fitting. One-to-one matching without replacement was then performed using a greedy nearest-neighbour algorithm on the logit of the propensity score, with a caliper width of 0.2× the standard deviation of the logit propensity score, as recommended by Austin [14]. Balance was assessed before and after matching using absolute standardized mean differences (|SMD|), with |SMD| < 0.20—and ideally < 0.10—considered indicative of adequate balance [15].

Long-term survival was estimated using the Kaplan–Meier method and compared between sexes with the log-rank test, both in the unmatched and the matched cohorts. Independent predictors of long-term all-cause mortality were assessed by multivariable Cox proportional hazards regression performed in the full unmatched cohort, including female sex, age, EuroSCORE II, LVEF, diabetes mellitus, renal impairment and a history of preoperative myocardial infarction. The proportional hazards assumption was examined by inspection of Schoenfeld residuals. Results are expressed as hazard ratios (HR) with 95% confidence intervals (CI).

A pre-specified subgroup analysis of long-term all-cause mortality by coronary disease pattern (single-vessel disease vs. multivessel disease with an LAD lesion) was performed using the Kaplan–Meier method with log-rank testing. Exact (Clopper–Pearson) 95% confidence intervals were reported for zero- and rare-event outcomes; Newcombe hybrid-score 95% confidence intervals were reported for corresponding between-group risk differences.

All statistical tests were two-sided, and a *p*-value < 0.05 was considered statistically significant. Analyses were performed using Python 3.14.5 with pandas 3.0.3, NumPy 2.4.6, SciPy 1.17.1, scikit-learn 1.8.0, and lifelines 0.30.3.s.

## 3. Results

### 3.1. Study Population and Baseline Characteristics

Between July 1999 and April 2025, 350 consecutive patients underwent MIDCAB at Hannover Medical School and were included in the analysis. Of these, 102 (29.1%) were female and 248 (70.9%) were male. The MIDCAB procedure was completed in all 350 patients without intraoperative conversion to conventional CABG. Single-vessel coronary artery disease involving the LAD was present in 227 patients (64.9%), whereas multivessel disease (in which MIDCAB was performed to address the LAD lesion) was present in 123 patients (35.1%). The median follow-up was 19.0 years (IQR 11.8–23.9), providing a long-term perspective on survival and graft-related outcomes.

Before matching, women were modestly older than men (65.1 ± 11.1 vs. 62.9 ± 11.0 years, *p* = 0.099) and had a higher EuroSCORE II (median 0.9 [IQR 0.8–1.3] vs. 0.8 [0.7–1.1], *p* = 0.004). Insulin-dependent diabetes mellitus was more prevalent in women (5.9% vs. 1.2%, *p* = 0.020), and there was a non-significant tendency toward higher rates of overall diabetes mellitus (23.5% vs. 14.9%, *p* = 0.076) and arterial hypertension (94.1% vs. 88.7%, *p* = 0.176) in the female group, while smoking was less frequent (22.5% vs. 33.1%, *p* = 0.068). LVEF, the distribution of single- versus multivessel disease, prior PCI, recent myocardial infarction, hyperlipidemia and renal impairment did not differ significantly between sexes. The baseline characteristics of the unmatched cohort are summarised in Table 1.

The overall risk profile of the cohort was low. The median age was 63 years (mean 64), median LVEF was 57% (mean 58%), 78.9% of procedures were elective, only 7.7% of patients had preoperative renal impairment, and preoperative dialysis was rare (0.9%). LVEF distribution by EuroSCORE II category was as follows: 303 of 350 patients (86.6%) had good LV function (LVEF > 50%), 47 (13.4%) had moderately reduced function (LVEF 31–50%), and no patient had severely reduced function (LVEF ≤ 30%); the corresponding numbers by sex were 92/102 women (90.2%) and 211/248 men (85.1%) with good LV function. No patient in either sex had documented moderate or greater mitral or tricuspid regurgitation. Acute coronary syndromes were present in only 7.1% of the cohort (STEMI 1.1%, NSTEMI 5.7%, recent MI 6.0%). These features collectively confirm the strongly selected, low surgical-risk nature of the cohort.

The absolute EuroSCORE II values in both sexes are low, reflecting this strongly selected low-risk nature of the MIDCAB cohort. This risk profile places both sexes near the mathematical floor of the EuroSCORE II scale, so that the female-sex component of the score (which contributes approximately 0.2 in isolation) is not fully expressed as an arithmetic difference between men and women at the group level, particularly once age and comorbidity have been closely matched.

### 3.2. Propensity Score Matching

**Figure 1:**
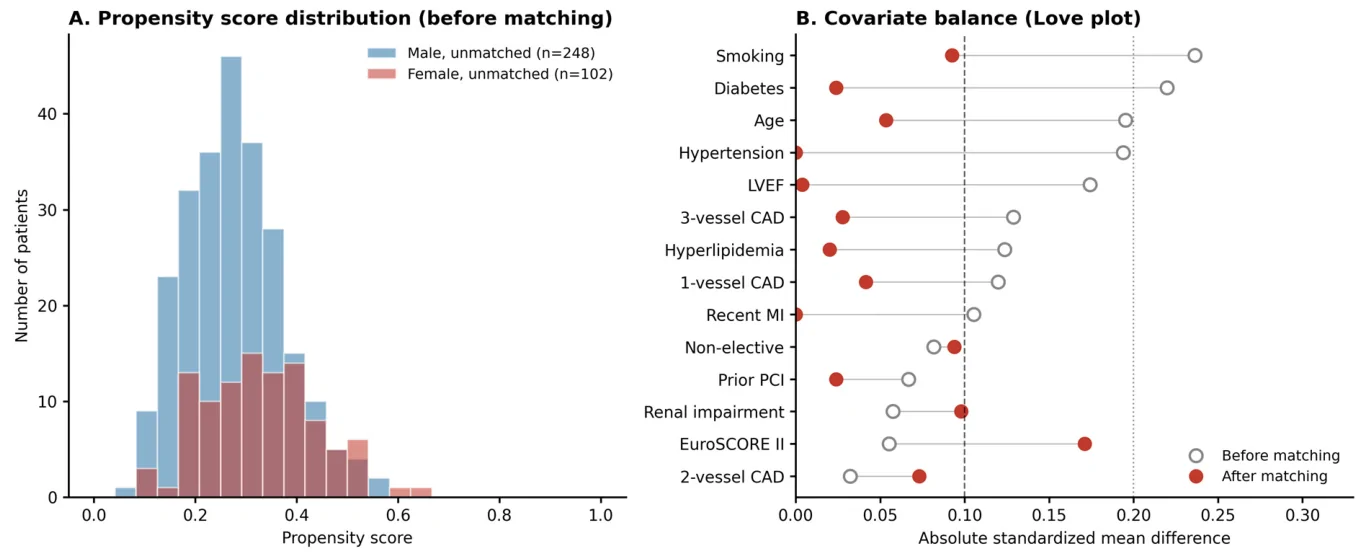
Propensity score distribution and covariate balance. (**A**) Histogram of estimated propensity scores in females (red) and males (blue) before matching, demonstrating substantial overlap. (**B**) Love plot of absolute standardized mean differences for all matching covariates before (open circles) and after (red circles) 1:1 propensity score matching. Dashed line: |SMD| = 0.10; dotted line: |SMD| = 0.20.

Greedy nearest-neighbour matching without replacement was performed with a caliper of 0.2 × SD of the logit propensity score (=0.106), as recommended by Austin [14]. A total of 100 of 102 women (98.0%) were successfully matched to a male counterpart, producing a final matched cohort of 200 patients (100 per group). The distribution of the estimated propensity scores demonstrated substantial overlap between sexes prior to matching (Figure 1A). After matching, all absolute standardized mean differences fell well below the conventional 0.20 threshold, with the majority below 0.10, indicating excellent covariate balance [15] (Figure 1B). Post-matching, women and men were comparable in age (64.9 ± 11.1 vs. 65.5 ± 11.5 years, *p* = 0.706), EuroSCORE II (median 0.9 [0.7–1.2] vs. 0.9 [0.7–1.1], *p* = 0.100), LVEF (58.2 ± 5.0% vs. 58.2 ± 5.6%, *p* = 0.979), and across all comorbid conditions and urgency categories (Table 2).

### 3.3. Intraoperative Outcomes

In the propensity-matched cohort, operative times were similar between women and men (126.4 ± 33.1 vs. 134.4 ± 37.2 min, *p* = 0.109). MIDCAB was performed as an off-pump procedure with a LIMA-to-LAD anastomosis in all cases. No intraoperative conversion to conventional CABG or cardiopulmonary bypass occurred in either group (0/200; exact 95% CI 0.00–1.83%); across the full 350-patient consecutive cohort, no conversion occurred either (0/350; exact 95% CI 0.00–1.05%). Intraoperative RBC transfusion requirements were comparable: 27.0% of women and 28.0% of men received at least one unit (*p* = 1.000), with similar median transfusion volumes (both 0 units; *p* = 0.852). Platelet transfusion was rarely required and did not differ between sexes (*p* = 0.802). Intraoperative outcomes are detailed in Table 3.

### 3.4. Postoperative In-Hospital Outcomes

Overall, the postoperative course was uneventful in the great majority of patients of both sexes. Thirty-day mortality was 0% in both groups. No patient developed postoperative stroke, and no patient required new postoperative renal replacement therapy. Postoperative myocardial infarction occurred in 1 woman (1.0%) and no men (*p* = 1.000). NOAF was infrequent and similar between groups (3.0% in women vs. 1.0% in men, *p* = 0.621). Re-exploration for bleeding was more frequent in women (5/100, 5.0%, exact Clopper–Pearson 95% CI 1.6–11.3%) than in men (0/100, 0.0%, 95% CI 0.0–3.6%); the Newcombe 95% CI for the between-sex risk difference was +0.3% to +11.2% (Fisher’s exact *p* = 0.059). Although the *p*-value did not reach the conventional 0.05 threshold in the matched cohort, the corresponding unmatched-cohort comparison (5/102, 4.9%, 95% CI 1.6–11.1% vs. 1/248, 0.4%, 95% CI 0.0–2.2%; risk difference +4.5%, Newcombe 95% CI +1.2 to +10.6%; Fisher’s exact *p* = 0.009) provides additional evidence that this represents a real signal rather than sampling noise, and it is discussed in detail in Section 4.2. Postoperative coronary angiography was indicated at some point during the 19-year median follow-up in 24.0% of women and 16.0% of men (*p* = 0.216).

The proportion of patients in whom postoperative coronary angiography was indicated reflects a cumulative estimate across the entire ~19-year median follow-up period rather than a peri-procedural rate. Indications for late coronary angiography during follow-up were clinically driven—most commonly recurrence or new onset of angina symptoms, new ischaemic changes on stress testing or imaging, and less frequently the investigation of unexplained heart failure or ventricular arrhythmia—but the specific indication for each individual angiogram was not recorded as a discrete coded field in our institutional database and cannot be enumerated in detail. Structured angiographic outcome documentation was available in a small subset: of 350 patients, 15 had a dated follow-up coronary angiogram with a structured LIMA-LAD assessment recorded (10 with good graft patency and 5 with poor or occluded graft); 2 patients required subsequent stenting of the LIMA-LAD anastomosis. For the remaining patients in whom postoperative angiography was indicated during follow-up, the corresponding structured outcome fields are empty, and results cannot be reported reliably. This limitation is acknowledged in Section 4.6.

**Figure 2:**
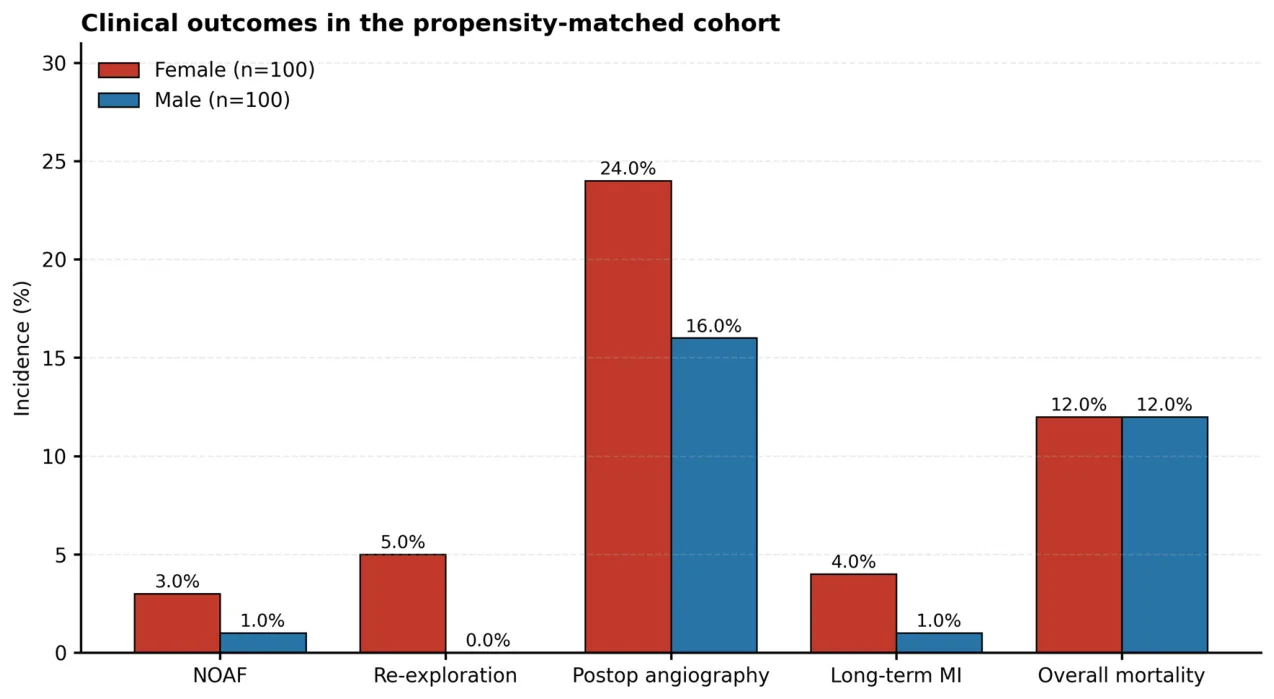
Incidence of key in-hospital and long-term clinical outcomes by sex in the propensity-matched cohort. All between-sex differences were non-significant. NOAF = new-onset atrial fibrillation; MI = myocardial infarction.

Length of ICU stay was short and clinically similar in both groups (median 1 day [IQR 1–2] in women vs. 1 day [5] in men, *p* = 0.075), as was the duration of mechanical ventilation (median 0 days in both groups, *p* = 0.078) and total hospital stay (median 8 days in both groups, *p* = 0.172). Peak postoperative myocardial markers—including creatine kinase, CK-MB and troponin—showed no significant between-group differences. Postoperative RBC transfusion in the ICU was slightly more frequent in women (mean 0.39 vs. 0.21 units, *p* = 0.044). Postoperative outcomes are summarised in Table 4 and visualised in Figure 2.

### 3.5. Long-Term Survival and Cardiac Outcomes

**Figure 3:**
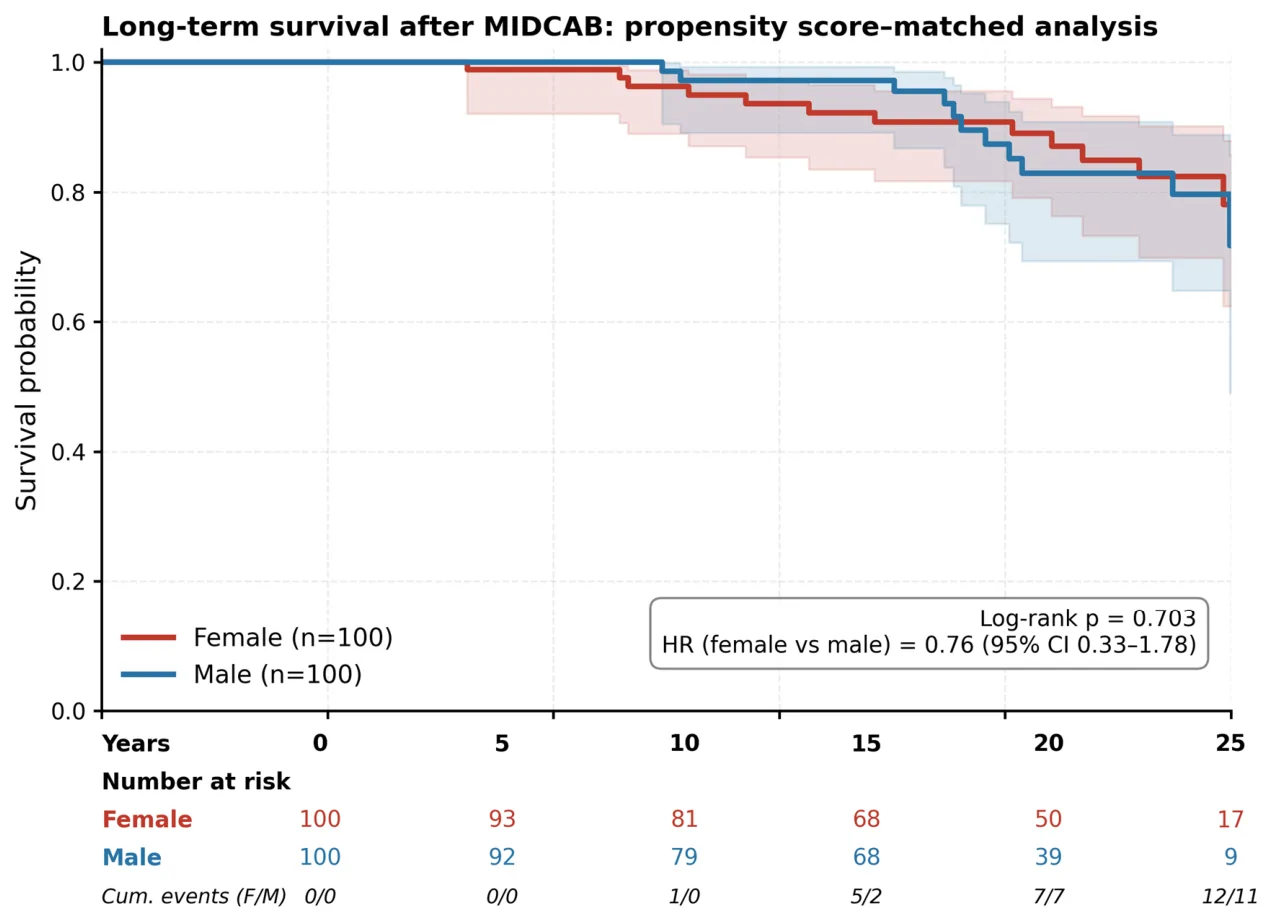
Kaplan–Meier curves for all-cause mortality stratified by sex in the propensity score-matched cohort (*n* = 200). Shaded regions represent 95% confidence intervals. The number-at-risk table and cumulative event counts are shown below the curves. Log-rank *p* = 0.703; adjusted hazard ratio for female sex 0.80 (95% CI 0.38–1.70).

Median follow-up was 19.9 years (IQR 11.8–24.5) in women and 18.6 years (11.8–23.9) in men (*p* = 0.197). During this extended follow-up period, all-cause mortality in the matched cohort was identical between sexes: 12 of 100 women (12.0%) and 12 of 100 men (12.0%) died (*p* = 1.000) (Table 5). The Kaplan–Meier estimates of long-term survival were superimposable, with no significant difference in survival functions between women and men (log-rank *p* = 0.703, Figure 3). Number-at-risk and cumulative event counts are presented within Figure 3.

Cause of death was documented for 33 of 35 deaths in the entire unmatched cohort. Only 4 deaths (12.1%) were classified as cardiac (terminal heart failure, myocardial infarction, or ventricular fibrillation), whereas 29 (87.9%) were of non-cardiac origin: stroke or intracerebral haemorrhage (7), malignancy (6), infection or respiratory failure including COVID-19 pneumonia (7), and other non-cardiac causes (9). This distribution supports the interpretation that the long-term mortality observed in this cohort was dominated by age-related competing causes rather than by cardiac events attributable to the surgical revascularization procedure. Documentation of cause of death was not available for 2 deaths (Section 4.6).

**Figure 4:**
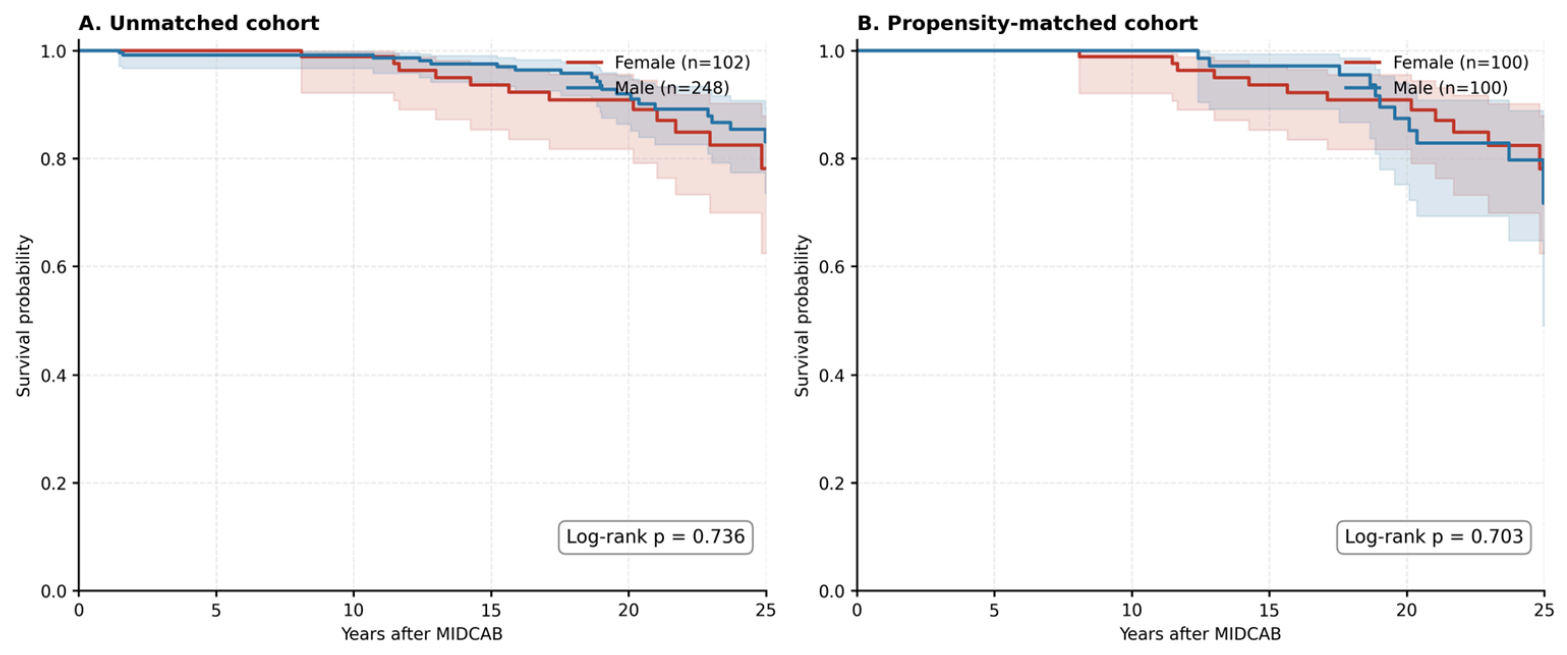
Kaplan–Meier survival curves for all-cause mortality in the unmatched cohort (*n* = 350). Shaded area: 95% confidence interval. No significant difference in long-term survival was observed between women and men (log-rank *p* = 0.736).

To confirm the robustness of these findings, Kaplan–Meier analysis was also performed in the full unmatched cohort, and it again yielded no significant survival difference between the sexes (log-rank *p* = 0.736; Figure 4). Long-term myocardial infarction occurred in 4 women (4.0%) and 1 man (1.0%) in the matched cohort (*p* = 0.369). Of patients in whom late coronary angiography was performed with structured LIMA-LAD documentation (*n* = 15 in the entire cohort), the LIMA-LAD anastomosis was patent and graded as good in 10 (66.7%) and occluded in 5 (33.3%); these results are descriptive given the small sample size and are further discussed in Section 4.3.

### 3.6. Multivariable Analysis of Predictors of All-Cause Mortality

**Figure 5:**
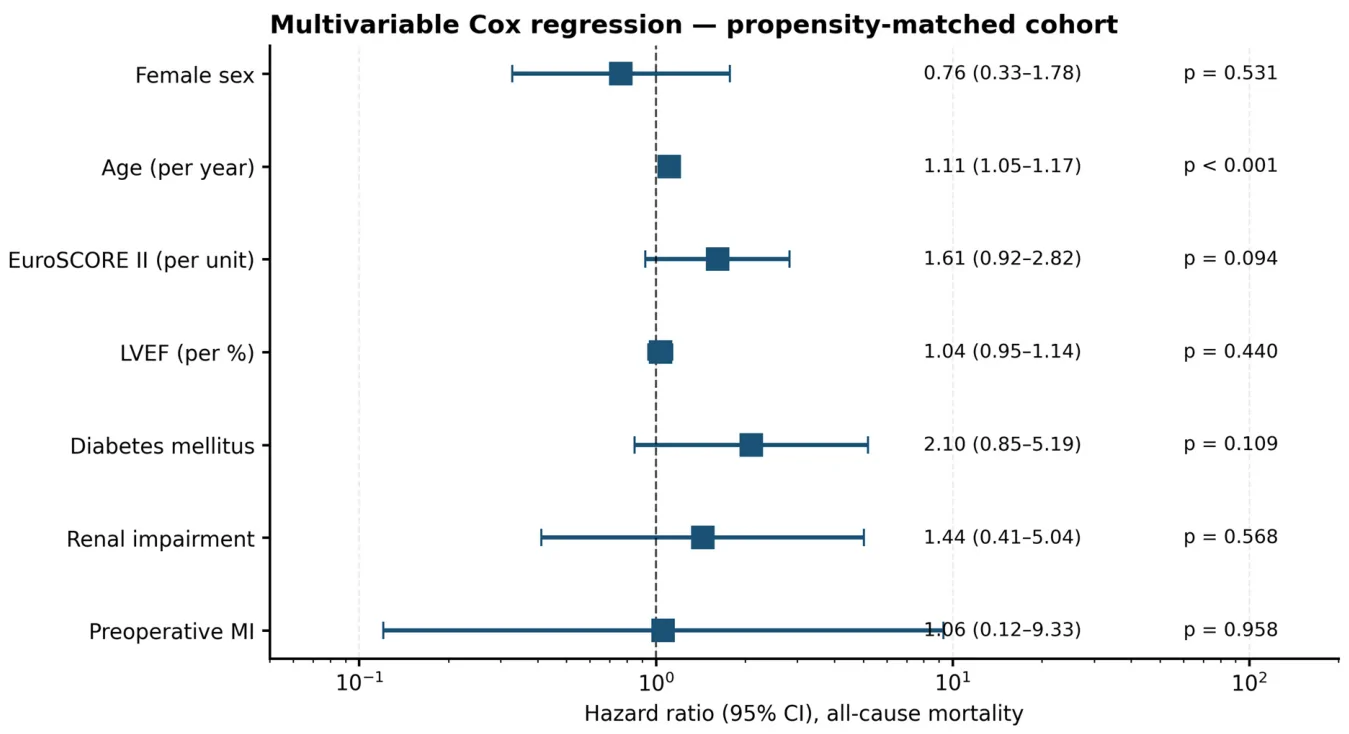
Forest plot of adjusted hazard ratios from the multivariable Cox proportional hazards regression for all-cause mortality in the full unmatched cohort. Squares represent point estimates; horizontal lines indicate 95% confidence intervals. The dashed vertical line marks HR = 1 (no effect). Age was the only strongly independent predictor of long-term mortality.

To identify independent predictors of long-term all-cause mortality and to formally adjust for baseline differences in risk factors, a multivariable Cox proportional hazards regression model was fitted in the full unmatched cohort (*n* = 349, after exclusion of a single patient with an artefactual EuroSCORE II value that was excluded from the Cox model for numerical stability). The model included female sex, age, EuroSCORE II, LVEF, diabetes mellitus, renal impairment and a history of preoperative myocardial infarction. After adjustment for these covariates, female sex was not associated with long-term mortality (adjusted HR 0.80, 95% CI 0.38–1.70, *p* = 0.560). Increasing age remained the only strongly independent predictor of mortality (HR 1.10 per year, 95% CI 1.05–1.15, *p* < 0.001). EuroSCORE II showed a borderline association with mortality (HR 1.67 per unit, 95% CI 1.00–2.82, *p* = 0.052), while LVEF, diabetes, renal impairment, and a history of preoperative myocardial infarction were not independently associated with the outcome. Results are presented in Table 6 and Figure 5.

### 3.7. Subgroup Analysis by Coronary Disease Pattern

A pre-specified subgroup analysis of long-term all-cause mortality by disease pattern was performed in the full unmatched cohort, including both isolated single-vessel LAD disease (SVD; *n* = 227) and LAD-predominant multivessel disease (MVD; *n* = 123, in whom MIDCAB was performed to address the LAD lesion). Twenty-year Kaplan–Meier survival was 92.3% in the SVD group and 90.9% in the MVD group, with no significant difference between them (log-rank *p* = 0.94). When stratified by sex within each subgroup, no significant sex-based mortality differences emerged: within the MVD subgroup, all-cause mortality was 10.0% in women (4/40) and 9.6% in men (8/83, log-rank *p* = 0.97); within the SVD subgroup, mortality was 12.9% in women (8/62) and 9.1% in men (15/165, log-rank *p* = 0.66).

## 4. Discussion

In this propensity score-matched analysis of 350 consecutive patients undergoing MIDCAB—a low-risk, appropriately selected population characterised by a median age of 63 years, median LVEF of 57%, elective status in 78.9%, acute coronary syndromes in only 7.1%, and no patient with LVEF ≤ 30% or moderate/severe valvular regurgitation—with a median follow-up approaching two decades, we observed three principal findings. First, MIDCAB carried extremely low perioperative risk in both sexes: 30-day mortality was 0%, and no patient developed perioperative stroke or required new postoperative dialysis. Second, perioperative and in-hospital outcomes—including operative time, transfusion requirements, NOAF, length of ICU and hospital stay, and peak postoperative cardiac biomarkers—were comparable between women and men in the matched cohort, with only a small excess of postoperative ICU transfusion in women and a signal of more re-exploration for bleeding in the female group. Third, after multivariable adjustment in the full unmatched cohort, female sex was not associated with long-term all-cause mortality; only advancing age emerged as a strongly independent predictor of late death. These observations contrast with the long-standing view that women are inherently disadvantaged after surgical coronary revascularization [3,4] and add to the growing body of evidence that, in selected operations and at experienced centres, sex-based outcome disparities may be substantially attenuated or eliminated [6,9,10,11,12]; however, these observations apply specifically to the low-risk MIDCAB population studied and should not be extrapolated to higher-risk surgical scenarios.

### 4.1. Female Sex and Conventional CABG: A Persistent Disparity

Female sex has historically been associated with worse outcomes after conventional CABG. In a recent single-centre series of 12,736 patients undergoing isolated CABG, women presented with more preoperative comorbidities, more frequent urgent or emergent operations, longer postoperative ICU stays and a higher risk of mortality than their male counterparts; female sex was confirmed as an independent risk factor for 30-day mortality (odds ratio 1.46, 95% CI 1.06–2.03) [3]. Analyses of the UK national adult cardiac surgery database have reached similar conclusions, with women experiencing higher rates of postoperative dialysis, deep sternal wound infection and longer hospital stay after CABG [4]. Consistent findings emerge from the large-scale California CABG Outcomes Reporting Program, in which women were at higher adjusted risk for operative mortality after CABG than men (odds ratio 1.61, 95% CI 1.40–1.84), despite adjustment for preoperative risk factors, and were also less likely to receive an internal mammary artery graft [16]. This consistent observation has resulted in the incorporation of female sex as an independent risk variable in EuroSCORE II [5].

Several explanations have been proposed for this disparity. Women undergoing CABG tend to be older, present with smaller coronary calibres that complicate technical performance, have a higher burden of diabetes and hypertension, and may receive less aggressive preventive cardiology and post-discharge therapy [3,4]. Importantly, several risk-adjusted analyses suggest that, once rigorous adjustment for these confounders is applied, the apparent excess risk in women is substantially attenuated—implying that a significant portion of the historical disparity reflects selection bias rather than an intrinsic biological disadvantage [6]. Notably, in a secondary analysis of the GOPCABE randomised trial of elderly patients undergoing CABG, female sex was not associated with 30-day mortality after multivariable adjustment, and conventional risk scores significantly overestimated mortality in elderly women [6].

### 4.2. Sex and MIDCAB: Converging Evidence

In the MIDCAB setting, evidence regarding sex-based outcomes has been considerably more limited. Gofus and colleagues, in a series of 384 patients followed at a single centre, reported longer surgical times, more frequent transfusion and higher rates of wound complications in women, but no significant difference in long-term mortality [9]. Friedrich and colleagues, in a propensity-matched analysis of 607 patients with single-vessel disease from the University Hospital Schleswig-Holstein, observed equivalent in-hospital outcomes and—after propensity matching—even superior long-term survival in women (*p* = 0.029) [10]. More recently, Zhao and colleagues, in a propensity-matched analysis of 471 patients with single-vessel disease, found no significant difference in in-hospital mortality, MACCE, perioperative myocardial infarction, stroke or reoperation for bleeding between the sexes, with wound complications and slightly longer hospital stay as the main attributes associated with female sex [11]. The most recent series—by Comanici and colleagues, a 20-year analysis of 676 patients with propensity matching—confirmed equivalent long-term survival between matched cohorts, with sex-specific rather than sex-disparate predictors of mortality [12]. Our findings—drawn from a cohort with a median follow-up approaching 20 years—are concordant with this emerging literature.

One observation in our data that deserves specific and honest discussion is the excess of re-exploration for bleeding in the female group. In the unmatched cohort, 5 of 102 women (4.9%, exact 95% CI 1.6–11.1%) required re-exploration for bleeding compared with 1 of 248 men (0.4%, exact 95% CI 0.0–2.2%), corresponding to a Newcombe 95% CI for the risk difference of +1.2% to +10.6% and a Fisher’s exact *p*-value of 0.009; in the matched cohort, the corresponding rates were 5/100 (5.0%) versus 0/100 (0.0%), risk difference +5.0% (Newcombe 95% CI +0.3% to +11.2%), Fisher’s exact *p* = 0.059. Although the matched-cohort comparison did not reach the conventional 0.05 threshold, the concordant direction and magnitude of the finding in both analyses suggests that this represents a real biological signal rather than sampling noise. Several plausible mechanisms are documented in the MIDCAB literature. First, the anatomically narrower and more constrained left-sided thoracotomy field in women can complicate LIMA harvest and haemostasis; second, smaller-calibre chest wall vessels are more susceptible to bleeding from the internal thoracic bed after LIMA takedown; third, the smaller mean body mass and circulating blood volume in women may lower the threshold at which a given absolute volume of bleeding produces haemodynamic instability prompting re-exploration; and fourth, a possible role of sex-related differences in platelet function has been described. Similar observations have been made by Gofus et al. [9] and by Zhao et al. [11]. Importantly, and reassuringly, this excess in re-exploration did not translate into any excess of perioperative death, stroke, dialysis, or long-term mortality; the equipoise in the primary safety and long-term survival endpoints therefore holds. Nevertheless, the finding merits attention in the operative planning of MIDCAB in women, and the prospective adjudication of the anatomical source of bleeding in future studies would be valuable.

A related consideration is the observed median hospital length of stay of 8 days in both sexes. Although shorter hospital stay is often cited as a benefit of MIDCAB relative to sternotomy CABG, an 8-day median is fully in line with the contemporary MIDCAB literature. Davierwala and colleagues, in the 20-year Leipzig experience (2667 patients), reported a median hospital stay of 8 days (IQR 7–10); Friedrich and colleagues reported a mean of 9.5–9.9 days [10]; and the most recent 20-year series by Comanici and colleagues reported a median of 8 days [12]. Our figure is therefore concordant with what other high-volume European MIDCAB centres publish and materially shorter than what is typically reported in conventional CABG series in comparable patient populations. In addition, the German healthcare system operates with hospital stay norms that are systematically longer than those in the UK and US healthcare systems, reflecting reimbursement structure, post-operative physiotherapy pathways, and the concentration of early cardiac rehabilitation within the acute admission rather than an ambulatory pathway. Importantly, length of stay in our cohort has decreased substantially over the 26-year study period. When patients are stratified by era of enrollment, the median hospital length of stay declined from 9 days (IQR 8–11) in 1999–2004 to 7 days (IQR 6–8) in every subsequent era (2005–2009, 2010–2014, 2015–2019, and 2020–2025 all had a median of 7 days). The aggregated median of 8 days across the whole cohort is therefore driven predominantly by the earliest years of the programme, when both institutional experience with MIDCAB and post-operative rehabilitation pathways were less mature. In the most recent five-year period (2020–2025), the median length of stay was 7 days (IQR 6–8), which more accurately reflects contemporary practice at our centre. Nevertheless, the median stay in our cohort remains longer than what is achievable in fast-track CABG programmes in the UK and US, and this dataset does not permit a direct within-institution comparison with contemporary sternotomy CABG length of stay, which is a limitation.

A further important consideration for a fair judgement of MIDCAB is the risk and consequences of intraoperative conversion to conventional sternotomy or cardiopulmonary bypass. In our consecutive series of 350 patients, no intraoperative conversion occurred (0/350; exact 95% CI 0.00–1.05%). In the largest published MIDCAB series, conversion occurs in approximately 1–3% of cases (Davierwala et al. reported 1.5% over 20 years; other series report 1–4%). The most common indications for conversion are inability to identify or dissect the LAD, haemodynamic instability during positioning or LAD occlusion, unexpected intrathoracic pathology, and technical inability to construct a satisfactory anastomosis. When conversion occurs, published outcomes are worse than for uncomplicated MIDCAB but comparable to what would be expected for a matched conventional CABG population and are substantially better than for aborted revascularization. The absence of any conversion in our cohort likely reflects both careful preoperative case selection by the heart team (Section 2.1) and the concentration of operative volume in an experienced minimally invasive surgical team.

The composition of our cohort—a mixture of isolated single-vessel LAD disease (64.9%) and LAD-predominant multivessel disease (35.1%)—warrants specific comment. Although one might a priori expect multivessel disease to confer a survival penalty relative to single-vessel disease, in our cohort this was not observed: 20-year Kaplan–Meier survival was 92.3% in the single-vessel subgroup and 90.9% in the multivessel subgroup, with no significant difference (log-rank *p* = 0.94). This concordance may reflect the dominance of the LIMA-LAD anastomosis as the physiologically most important long-term graft, and the fact that residual non-LAD disease in this cohort was addressed either medically or by staged percutaneous coronary intervention as part of a hybrid strategy. Our institutional database does not capture whether the non-LAD territory in multivessel patients was addressed by staged PCI or managed medically, and this residual heterogeneity is discussed in the Limitations section (Section 4.6).

In terms of perioperative safety, we observed no significant excess of major perioperative morbidity in women undergoing MIDCAB. While there was a signal of more re-exploration for bleeding and a slight excess in postoperative RBC transfusion (mean 0.39 vs. 0.21 units, *p* = 0.044) in women, no patient in either group experienced perioperative death, stroke or new dialysis. Operative times in our series were comparable to those reported by Friedrich et al. [10] and did not differ between sexes. The absence of intraoperative conversion to sternotomy in any patient indicates that, when patient selection and surgical experience are appropriate, MIDCAB can be performed safely irrespective of sex.

Our long-term mortality data provide unique reassurance. With a median follow-up of approximately 19 years—among the longest reported for any MIDCAB series, comparable to the 20-year follow-up achieved by Manuel and colleagues [8] and the recent series by Comanici and colleagues [12]—all-cause mortality was identical between matched women and men (12.0% in each group), and the multivariable-adjusted hazard ratio for female sex in the full unmatched cohort was 0.80 (95% CI 0.38–1.70), implying no clinically meaningful sex-based excess risk.

### 4.3. Why MIDCAB May Be Particularly Suited to Women

Several physiological and technical considerations may explain why women—historically at higher risk after conventional CABG—appear to fare as well as men after MIDCAB. The avoidance of median sternotomy abolishes the small but real risk of deep sternal wound infection and dehiscence, complications to which women, particularly those with diabetes, obesity, or large breasts, have traditionally been more susceptible [4]. The avoidance of cardiopulmonary bypass eliminates the systemic inflammatory response, reduces neurocognitive injury, and decreases transfusion requirements—all of which may disproportionately benefit smaller-statured patients with lower circulating blood volume. The published literature report high LIMA-LAD long-term patency (exceeding 90% at 10–15 years) across multiple series [1,2] independent of sex, providing an expected mechanism for durable protection of the LAD territory in both women and men.

### 4.4. Age as the Dominant Predictor

In multivariable Cox regression, age was the only strongly independent predictor of long-term mortality (HR 1.10 per year, *p* < 0.001), with EuroSCORE II showing a borderline association (HR 1.67 per unit, *p* = 0.052). This pattern underscores that, in patients selected for and surviving MIDCAB, late mortality is determined primarily by aging-related competing causes rather than by sex, ventricular function, or coronary disease pattern. This interpretation is directly supported by the cause-of-death data in our cohort (Section 3.5): of 33 deaths with documented cause, only 4 (12%) were classified as cardiac, whereas 29 (88%) were of non-cardiac origin—predominantly stroke, malignancy, and infection/respiratory failure. This pattern aligns with reports from Manuel and colleagues, who demonstrated that long-term survival after MIDCAB approximates that of the age-matched general population, with a standardised mortality ratio of 0.94 in single-vessel disease [8]. In this context, the observation of low absolute EuroSCORE II values in both sexes should be interpreted as consistent with, rather than in contradiction of, the score’s known sex-specific weighting: the female-sex component of EuroSCORE II contributes approximately 0.2 in isolation, but once age, LVEF, urgency, and comorbidity have been matched between women and men (as in our propensity-matched cohort), the residual sex contribution is expressed within a very low baseline and, at the group level, is not fully arithmetically evident. These observations apply specifically to a low-risk, appropriately selected MIDCAB population and should not be extrapolated to higher-risk clinical scenarios.

### 4.5. Clinical Implications

Our findings carry several practical implications. First, in patients with isolated LAD disease or LAD-predominant multivessel disease in whom hybrid revascularization is planned and who fulfil the selection criteria described in Section 2.1, MIDCAB should be offered to women on the same indications, and with the same expectations of outcome, as to men. Second, although the inclusion of female sex as an independent risk variable in EuroSCORE II [5] is appropriate for conventional CABG, it may overestimate operative risk in women undergoing MIDCAB in a comparably low-risk population; risk-prediction models may need to be re-calibrated for minimally invasive coronary surgery, as previously noted in the elderly by Faerber and colleagues [6]. Third, the findings of the present study apply specifically to the low-risk, appropriately selected MIDCAB population that entered the cohort. They should not be extrapolated to older women, frail women, or women with substantial comorbidity, in whom the choice between MIDCAB, conventional sternotomy CABG, and percutaneous revascularization must be individualised and in whom the outcomes of MIDCAB have not been evaluated in the present study. Contemporary guidance from the 2018 ESC/EACTS guidelines on myocardial revascularization [13] should continue to inform this individualised decision-making.

### 4.6. Limitations

Several limitations of this study should be acknowledged. First, the analysis is retrospective and single-centre; despite the use of propensity score matching, residual confounding from unmeasured variables (for example, body surface area, frailty indices, preoperative haematocrit, medications, and socioeconomic factors) cannot be excluded. NYHA functional class, body mass index, alcohol-use history, and formal heart failure classification were not systematically recorded as discrete fields in the institutional database across the study period and could therefore not be included as covariates in the propensity model or the multivariable Cox model. Second, the relatively small number of female patients (*n* = 102) limits the statistical power to detect modest sex-based differences in low-frequency events such as perioperative stroke, dialysis or postoperative myocardial infarction, all of which were rare in our cohort. Third, the matched analysis required the exclusion of two unmatched female patients (98.0% matching rate); this minor loss of data is unlikely to have biased the findings. Fourth, the observed excess of re-exploration for bleeding in women (5.0% vs. 0.0%) deserves further investigation in larger cohorts, as previously highlighted in other MIDCAB series [9]. Although this finding is directionally consistent across both the unmatched (Fisher’s exact *p* = 0.009) and matched (*p* = 0.059) cohorts, the specific anatomical source of bleeding (LIMA harvest bed, thoracotomy incision, or LAD anastomosis) was not systematically captured in our operative record, precluding definitive causal adjudication. Fifth, late coronary angiography was performed in a minority of patients (*n* = 15 with structured documentation), precluding firm conclusions about sex-specific LIMA-LAD patency; furthermore, the indications for individual late angiograms and the results for the larger group in whom angiography was indicated during follow-up were not systematically captured as discrete fields in the institutional database. Sixth, cause-specific mortality (cardiac vs. non-cardiac) could not be reliably ascertained for all patients given the very long follow-up; however, cause of death was documented for 33 of 35 deaths (94.3%), and the resulting distribution (12% cardiac vs. 88% non-cardiac) supports the interpretation that the long-term mortality observed in this cohort was dominated by age-related competing causes rather than cardiac events attributable to the procedure. Seventh, the cohort included both single-vessel disease (64.9%) and LAD-predominant multivessel disease (35.1%). Our database does not capture whether residual non-LAD disease in multivessel patients was addressed by staged percutaneous coronary intervention as part of a hybrid strategy or managed medically. This residual heterogeneity, and the absence of a discrete field for completeness of revascularization, is acknowledged as a limitation of the retrospective design. Eighth, EuroSCORE II was calculated retrospectively for patients operated before its 2012 publication using the prospectively recorded preoperative variables; although this is standard practice for retrospective cohort studies with extended follow-up, it introduces the possibility of subtle miscalibration for the earliest patients. Ninth, enrollment was concentrated in the earlier years of the 26-year study period (see Section 4.7 and Supplementary Table S1), with a marked reduction in annual MIDCAB volume after 2010. Propensity score matching was performed across the entire cohort without stratification by era; residual era-related confounding cannot therefore be excluded, and the applicability of our findings to contemporary MIDCAB practice should be interpreted with this in mind. Tenth, the population studied was one at low surgical risk (median age 65 years, median LVEF 58%, 7% acute coronary syndromes, no LVEF ≤ 30%, no moderate/severe valvular disease), and the applicability of our findings to higher-risk MIDCAB candidates has not been evaluated. Finally, all procedures were performed at a single tertiary centre by an experienced minimally invasive cardiac surgical team, and generalisability to lower-volume centres should be made with caution.

### 4.7. Temporal Trends in Enrollment

As detailed in Supplementary Table S1, the annual MIDCAB caseload at our institution was concentrated in the earlier years of the 26-year enrollment period, with 130 procedures in 1999–2004, 103 in 2005–2009, 34 in 2010–2014, 38 in 2015–2019, and 44 in 2020–2025. Peak enrollment occurred in 2000–2001 (45 procedures per year); after 2010, the annual caseload has typically ranged between 3 and 14 procedures per year. The principal reasons for this progressive contraction are: (i) the expansion of drug-eluting stent technology and refinement of PCI techniques for isolated LAD disease over the 2000s, which has moved the multidisciplinary heart team’s threshold for surgical LAD-only revascularization progressively upward; (ii) the corresponding rise of hybrid revascularization strategies in which PCI-first pathways are favoured for anatomically borderline non-LAD lesions; (iii) evolving institutional referral patterns, with an increasing proportion of complex multivessel-disease cases referred for full sternotomy CABG rather than for hybrid MIDCAB; and (iv) the general contraction of isolated single-vessel surgical revascularization across contemporary German cardiac surgical practice. This temporal contraction is not unique to our institution and is broadly consistent with trends reported in the recent MIDCAB literature. The resulting era heterogeneity is acknowledged as a limitation in Section 4.6.

## 5. Conclusions

In this propensity score-matched analysis of patients undergoing MIDCAB with up to two decades of follow-up in a low-risk, appropriately selected population, female sex was not associated with adverse perioperative or long-term outcome. Thirty-day mortality was 0% in both women and men, major perioperative complications were rare and equally distributed, and long-term all-cause mortality was identical between matched sexes. After multivariable adjustment in the full unmatched cohort, only advancing age—and not female sex, ventricular function, diabetes, or renal impairment—emerged as an independent predictor of long-term mortality. These results support MIDCAB as a safe, durable and sex-neutral revascularization strategy for carefully selected low-risk patients with single-vessel and LAD-predominant coronary artery disease, and call into question the routine inclusion of female sex as an independent risk factor in this minimally invasive surgical setting. These conclusions should not be extrapolated to higher-risk MIDCAB candidates in whom the outcomes of MIDCAB have not been evaluated in the present study.

## Figures and Tables

**Table 1:** Baseline characteristics of the entire MIDCAB cohort before propensity score matching (*n* = 350).

Variable	Female (*n* = 102)	Male (*n* = 248)	*p*-Value	SMD
Age, years	65.1 ± 11.1	62.9 ± 11.0	0.099	0.195
EuroSCORE II	0.9 (0.8–1.3)	0.8 (0.7–1.1)	0.004	−0.055
LVEF, %	58.1 ± 5.0	57.1 ± 5.8	0.116	0.174
LVEF category (per EuroSCORE II)				
Good (>50%)	92 (90.2%)	211 (85.1%)		
Moderate (31–50%)	10 (9.8%)	37 (14.9%)		
Poor (≤30%)	0 (0.0%)	0 (0.0%)		
(Overall LVEF category *p*-value)			0.269	
Moderate/severe MR (MVI > II)	0 (0.0%)	0 (0.0%)	1.000	-
Moderate/severe TR (TVI > II)	0 (0.0%)	0 (0.0%)	1.000	-
Urgency status, *n* (%)				
Elective	78 (76.5%)	198 (79.8%)		
Urgent	20 (19.6%)	41 (16.5%)		
Emergency	4 (3.9%)	9 (3.6%)		
(Overall urgency *p*-value)			0.774	
Single-vessel disease (LAD)	62 (60.8%)	165 (66.5%)	0.368	−0.120
Two-vessel disease	24 (23.5%)	55 (22.2%)	0.893	0.032
Three-vessel disease	16 (15.7%)	28 (11.3%)	0.342	0.129
Recent MI (<90 days)	8 (7.8%)	13 (5.2%)	0.494	0.105
STEMI	1 (1.0%)	3 (1.2%)	1.000	−0.022
NSTEMI	7 (6.9%)	13 (5.2%)	0.734	0.068
Prior PCI	23 (22.5%)	63 (25.4%)	0.669	−0.067
Smoking	23 (22.5%)	82 (33.1%)	0.068	−0.236
Diabetes (any)	24 (23.5%)	37 (14.9%)	0.076	0.220
Non-insulin-dependent DM	18 (17.6%)	34 (13.7%)	0.438	0.108
Insulin-dependent DM	6 (5.9%)	3 (1.2%)	0.020	0.255
Arterial hypertension	96 (94.1%)	220 (88.7%)	0.176	0.194
Hyperlipidemia	48 (47.1%)	132 (53.2%)	0.352	−0.124
Renal impairment	9 (8.8%)	18 (7.3%)	0.781	0.058
Preoperative dialysis	0 (0.0%)	3 (1.2%)	0.559	−0.156

Values are mean ± SD, median (IQR) or *n* (%). *p*-values from Welch’s *t*-test, Mann–Whitney U, χ^2^ or Fisher’s exact test as appropriate. SMD = standardized mean difference; |SMD| < 0.10 indicates negligible imbalance. Renal impairment defined as estimated glomerular filtration rate < 60 mL/min/1.73 m^2^ by CKD-EPI or dependence on chronic dialysis. LVEF category as defined by EuroSCORE II. DM = diabetes mellitus; LAD = left anterior descending artery; LVEF = left ventricular ejection fraction; MI = myocardial infarction; MR = mitral regurgitation; PCI = percutaneous coronary intervention; TR = tricuspid regurgitation.

**Table 2:** Baseline characteristics of the propensity score-matched cohort (*n* = 200; 100 female–male pairs).

Variable	Female (*n* = 100)	Male (*n* = 100)	*p*-Value	SMD
Age, years	64.9 ± 11.1	65.5 ± 11.5	0.706	−0.053
EuroSCORE II	0.9 (0.7–1.2)	0.9 (0.7–1.1)	0.100	0.171
LVEF, %	58.2 ± 5.0	58.2 ± 5.6	0.979	−0.004
Single-vessel disease (LAD)	62 (62.0%)	64 (64.0%)	0.884	−0.041
Two-vessel disease	23 (23.0%)	20 (20.0%)	0.731	0.073
Three-vessel disease	15 (15.0%)	16 (16.0%)	1.000	−0.028
Recent MI (<90 days)	6 (6.0%)	6 (6.0%)	1.000	0.000
Prior PCI	23 (23.0%)	22 (22.0%)	1.000	0.024
Smoking	23 (23.0%)	27 (27.0%)	0.624	−0.092
Diabetes (any)	23 (23.0%)	22 (22.0%)	1.000	0.024
Arterial hypertension	94 (94.0%)	94 (94.0%)	1.000	0.000
Hyperlipidemia	47 (47.0%)	48 (48.0%)	1.000	−0.020
Renal impairment	9 (9.0%)	12 (12.0%)	0.645	−0.098
Preoperative dialysis	0 (0.0%)	2 (2.0%)	0.497	−0.202
Elective status	78 (78.0%)	74 (74.0%)	0.617	0.093

After matching, all standardized mean differences were below 0.20, indicating adequate balance. Statistical conventions as in Table 1.

**Table 3:** Intraoperative outcomes in the propensity score-matched cohort.

Variable	Female (*n* = 100)	Male (*n* = 100)	*p*-Value
Operative time, min	126.4 ± 33.1	134.4 ± 37.2	0.109
Intraoperative RBC transfusion (≥1 unit), *n* (%)	27 (27.0%)	28 (28.0%)	1.000
Intraoperative conversion to CABG, *n* (%)	0 (0.0%)	0 (0.0%)	1.000

Continuous variables are mean ± SD or median (IQR); categorical variables are *n* (%). RBC = red blood cell.

**Table 4:** Postoperative in-hospital outcomes in the propensity score–matched cohort.

Variable	Female (*n* = 100)	Male (*n* = 100)	*p*-Value
ICU length of stay, days	1.0 (1.0–2.0)	1.0 (1.0–1.0)	0.075
Hospital length of stay, days	8.0 (7.0–10.0)	8.0 (7.0–9.0)	0.172
Max postoperative CK-MB, U/L	35.0 (24.8–41.0)	32.5 (26.0–41.0)	0.685
Max postoperative CK, U/L	425.5 (356.0–566.0)	485.0 (363.8–696.2)	0.263
Max postoperative troponin, ng/L	28.0 (20.8–35.0)	28.0 (22.0–37.2)	0.529
New-onset atrial fibrillation	3 (3.0%)	1 (1.0%)	0.621
Re-exploration for bleeding	5 (5.0%)	0 (0.0%)	0.059
New postoperative dialysis	0 (0.0%)	0 (0.0%)	1.000
Postoperative stroke	0 (0.0%)	0 (0.0%)	1.000
Postoperative MI	1 (1.0%)	0 (0.0%)	1.000
Postop angiography (indicated during follow-up)	24 (24.0%)	16 (16.0%)	0.216

Values are median (IQR) for continuous variables and *n* (%) for categorical variables. Peak troponin refers to peak postoperative high-sensitivity troponin T measured by the institutional laboratory. Postoperative angiography rate reflects cumulative angiographic evaluation over the ~19-year median follow-up and is not a peri-procedural rate. CK = creatine kinase; ICU = intensive care unit; MI = myocardial infarction.

**Table 5:** Long-term follow-up outcomes in the propensity score–matched cohort.

Variable	Female (*n* = 100)	Male (*n* = 100)	*p*-Value
Follow-up, years	19.9 (11.8–24.5)	18.6 (11.8–23.9)	0.197
30-day mortality	0 (0.0%)	0 (0.0%)	1.000
All-cause mortality (overall)	12 (12.0%)	12 (12.0%)	1.000
Long-term MI	4 (4.0%)	1 (1.0%)	0.369

Thirty-day mortality is defined as death within 30 days of surgery. Median follow-up exceeded 18 years in both groups.

**Table 6:** Multivariable Cox proportional hazards regression for all-cause mortality in the full unmatched cohort (*n* = 349).

Variable	Adjusted HR	95% CI	*p*-Value
Female sex	0.80	0.38–1.70	0.560
Age (per year)	1.10	1.05–1.15	<0.001
EuroSCORE II (per unit)	1.67	1.00–2.82	0.052
LVEF (per %)	0.98	0.92–1.05	0.574
Diabetes mellitus	1.76	0.80–3.86	0.160
Renal impairment	1.57	0.49–5.04	0.449
Preoperative MI history	0.63	0.08–5.01	0.665

Outcome: all-cause mortality in the full unmatched cohort (*n* = 349 after exclusion of a single EuroSCORE II data-entry outlier). Female sex was not independently associated with long-term mortality after multivariable adjustment. Age was the only strongly independent predictor. HR = hazard ratio; CI = confidence interval; LVEF = left ventricular ejection fraction; MI = myocardial infarction.

## Data Availability

The data presented in this study are available on reasonable request from the corresponding author. The data are not publicly available owing to privacy and ethical restrictions.

## Supplementary Materials

The following supporting information can be downloaded at: https://www.mdpi.com/article/10.3390/jcm15176821/s1, Table S1: Number of MIDCAB procedures per year at Hannover Medical School, stratified by sex (July 1999–April 2025; *n* = 350).
