# Supplementary material for: Sex Disparities in Outcomes After Minimally Invasive Direct CABG for Single-Vessel Disease: A Propensity Score-Matched Analysis
Source: J Clin Med. 2026 Sep 3;15(17):6821. doi: 10.3390/jcm15176821 (PMC13566088; doi:10.3390/jcm15176821)
Supplement: Supplementary file 1 [file jcm-15-06821-s001.zip › jcm-4452661-supplementary.pdf]

Journal of Clinical Medicine

MDPI

Supplementary Table

Supplementary Table S1. Number of MIDCAB procedures per year at Hannover Medical School, stratified by sex (July 1999 – April 2025; n = 350).

Year	Female	Male	Total
1997	1	0	1
1999	0	2	2
2000	16	29	45
2001	12	33	45
2002	5	7	12
2003	2	3	5
2004	8	13	21
2005	6	15	21
2006	5	17	22
2007	7	22	29
2008	4	16	20
2009	1	10	11
2010	1	4	5
2011	2	1	3
2012	0	1	1
2013	5	9	14
2014	5	6	11
2015	1	4	5
2016	3	4	7
2017	3	10	13
2018	4	4	8
2019	2	3	5
2020	1	9	10
2021	1	5	6
2022	1	2	3
2023	4	7	11
2024	0	3	3
2025	2	9	11
Total 1999–2004	43	87	130
Total 2005–2009	23	80	103
Total 2010–2014	13	21	34
Total 2015–2019	13	25	38
Total 2020–2025	9	35	44
Overall total	102	248	350

Annual and 5-year enrolment counts by sex. Note the concentration of caseload in 1999–2009 (66.6% of total) and progressive contraction after 2010, attributable to expansion of drug-eluting stent PCI for isolated LAD disease, evolving referral patterns, and the general contraction of isolated single-vessel surgical revascularization in contemporary German practice (see Section 4.7).
